# Identification of Quantitative Trait Loci Controlling the Development of Prickles in Eggplant by Genome Re-sequencing Analysis

**DOI:** 10.3389/fpls.2021.731079

**Published:** 2021-09-08

**Authors:** Zongwei Qian, Bin Zhang, Haili Chen, Lei Lu, Mengqi Duan, Jun Zhou, Yanling Cui, Dayong Li

**Affiliations:** ^1^National Engineering Research Center for Vegetables, Beijing Vegetable Research Center, Beijing Academy of Agriculture and Forestry Science, Beijing, China; ^2^Beijing Key Laboratory of Vegetable Germplasm Improvement, Beijing, China; ^3^Key Laboratory of Biology and Genetic Improvement of Horticultural Crops (North China), Ministry of Agriculture and Rural Affairs of the P. R. China, Beijing, China; ^4^College of Life Science and Technology, Jining Normal University, Ulanqab, China; ^5^Turf Research Institute, Beijing Forestry University, Beijing, China; ^6^College of Life Sciences, Shandong Normal University, Jinan, China

**Keywords:** eggplant, prickle, calyx, QTL mapping, WUSCHEL-related homeobox protein

## Abstract

Eggplant (*Solanum melongena* L.) is the third most important crop in the family of Solanaceae. Prickles are considered as the undesirable traits during the plantation of eggplant and the transportation of fruits. In this study, we constructed a high-quality genetic linkage Bin map derived from the re-sequencing analysis on a cross of a prickly wild landrace, 17C01, and a cultivated variety, 17C02. The major quantitative trait locus (QTL) controlling the development of prickles on the calyx (explained 30.42% of the phenotypic variation), named as *qPC.12*, was identified on a ~7 kb region on chromosome 12. A gene within *qPC.12*, which encodes a WUSCHEL-related homeobox-like protein, with higher expression levels in 17C01 calyx and 22-bp deletion in 17C02 was probably the functional gene for prickle formation. Results from this study would ultimately facilitate uncovering the molecular regulatory mechanisms underlying the development of a prickle in eggplant.

## Introduction

Eggplant (*Solanum melongena* L.), of the Solanaceae family, is a common vegetable in Africa, Asia, and Southern Europe (Polignano et al., 2010). Among solanaceous crops, eggplant is the third most important crop following *Solanum tuberosum* (potato) and *Solanum lycopersicum* (tomato) with a total global production of 52 million tons in 2017 (FAOSTAT 2017; http://faostat.fao.org). In the past decade, the production of eggplant has experienced a faster increase than that of potato and tomato (FAOSTAT 2017; http://faostat.fao.org). It becomes increasingly important to develop new eggplant varieties with high-yielding and improved agronomic traits such as optimum plant architecture and fruit shape, minimum risk of deterioration during transport, and longer shelf time.

Wild eggplant and cultivated varieties have significant phenotypic divergence in morphological traits, such as leaf morphology, fruit size, shape and color, and plant architecture. Among these traits, the “prickly phenotype” is significant in eggplant breeding. In eggplant with the prickly phenotype, abundant prickles are visible on various tissues, including the stem, leaf, petiole, calyx, and fruit stalk. Cultivated eggplant varieties usually have much fewer prickles than wild types. However, prickles on the calyx are still commonly found in many varieties, despite the prickles on the stem, leaf, petiole, and fruit stalk are generally absent as the result of the improved breeding and cultivation techniques. Breeding new eggplant varieties with non-prickly calyx requires the characterization of the molecular and genetic mechanisms underlying this complex agronomic trait, while the map-based gene cloning based on the large-scale molecular markers and high-quality genetic maps become indispensable for such task.

The first genetic map associated with fruit shape and color in eggplant was constructed using randomly amplified polymorphic DNA (RAPD) and amplified fragment length polymorphism (AFLP) markers (Nunome et al., 2001). Since then, several genetic linkage maps were constructed for disease resistance, parthenocarpy trait, and plant morphological traits using simple sequence repeat (SSR), single nucleotide polymorphisms (SNPs), RAPD, and AFLP markers in eggplant (Barchi et al., 2010, 2012, 2018; Lebeau et al., 2013; Frary et al., 2014; Miyatake et al., 2016; Wei et al., 2020). The identification of quantitative trait locus (QTL) associated with several agronomic traits has been developed in eggplant along with the improvement of the construction of genetic linkage maps. For example, a number of QTLs have been identified using an intraspecific F2 population and a 238-loci linkage map for anthocyanin pigmentation, fruit morphology (e.g., weight, length, diameter, metabolic content, and shape), and prickless (Doganlar et al., 2002; Barchi et al., 2010, 2012; Portis et al., 2014; Toppino et al., 2016). However, the identification and characterization of QTLs and functional genes underlying important agronomic traits in eggplant have largely lagged behind when compared to other vegetable crops, such as tomato and cucumber, partly due to the lack of a genetic linkage map with high-density markers. With the advances in the next-generation sequencing (NGS) technologies, four eggplant reference genomes were published at present (Hirakawa et al., 2014; Barchi et al., 2019; Wei et al., 2020; Li et al., 2021), which would greatly facilitate developing a large number of SNP markers for genetic map construction, leading to the improved efficiency of fine gene mapping.

The prickle is formed by the deformation of trichome and cortical cells. The gene regulatory network of the development of trichome has been thoroughly investigated in *Arabidopsis thaliana*. A central trimeric complex formed by GLABRA1 (GL1), GLABRA3/ENHANCER of GL3 (GL3/EGL3), and TRANSPARENT TESTA GLABRA1 (TTG1), which belong to the homeodomain-leucine zipper (HD-ZIP) proteins, plays key roles as positive factors in the initiation of *A. thaliana* trichome (Oppenheimer et al., 1991; Walker et al., 1999; Payne et al., 2000; Zhang and Oppenheimer, 2004). This complex may activate the expression of another homeodomain protein GLABRA2 (GL2) (Grebe, 2012; Pattanaik et al., 2014). In addition, several R3 MYB factors, such as TRIPTYCHON (TRY), CAPRICE (CPC), ENHANCE OF TRY AND CPC1, 2, and 3 (ETC1, ETC2, and ETC3), CPC-LIKE MYB3 (CPL3), TRICHOMELESS1 (TCL1), and TRICHOMELESS2 (TCL2), were reported as negative factors during trichome formation by disrupting the function of the trimeric complex (Wada et al., 1997; Schellmann et al., 2002; Kirik et al., 2004a,b; Wang and Chen, 2014). In horticultural crops, some HD-ZIP proteins were also identified to participate in the development regulation of multicellular trichomes, such as Glabrous 1 (CsGL1), Micro-trichome (Mict), TRICHOME-LESS/Glabrous 3 (TRIL/CsGL3) and *Cucumis sativus* TRANSPARENT TESTA GLABRA1 (CsTTG1) in *C. sativus* (cucumber) and *CsWUS* in citrus (Zhao et al., 2015; Cui et al., 2016; Liu et al., 2016; Chen et al., 2017; Zhang et al., 2020a). Several development-related transcription factors such as bHLH, C2H2, MYB, TCP, and WRKY, which were specifically downregulated or upregulated in developing prickles, were also found in eggplant (Zhang et al., 2021).

For prickly phenotype in horticultural crops, there are several reports on eggplant, rose, and citrus by genetic map construction and QTL analysis, but many were insufficient for delineating the key functional genes (Frary et al., 2014; Gramazio et al., 2014; Portis et al., 2015; Miyatake et al., 2020; Zhou et al., 2020). Miyatake et al. (2020) identified a *Pl* locus conferring the absence of prickles on chromosome 6 using a linkage map of the F2 population (Miyatake et al., 2020). In this study, we constructed a high-density genetic linkage Bin map derived from the F2 population of 17C01, a prickly wild landrace as the female parent, and 17C02, a cultivated variety as the male parent with much fewer prickles. We further identified the major QTLs associated with the abundance of prickles on the stem, leaf, petiole, and calyx. Finally, we proposed a gene, encoding a putative WUSCHEL-related homeobox 3B protein, as the candidate gene responsible for the development of prickle on the calyx. These results would pave the way to characterize the molecular mechanism of the development of prickle in eggplant and to facilitate breeding new non-prickly eggplant varieties.

## Materials and Methods

### Plant Materials and Growth Condition

**Figure 1:**
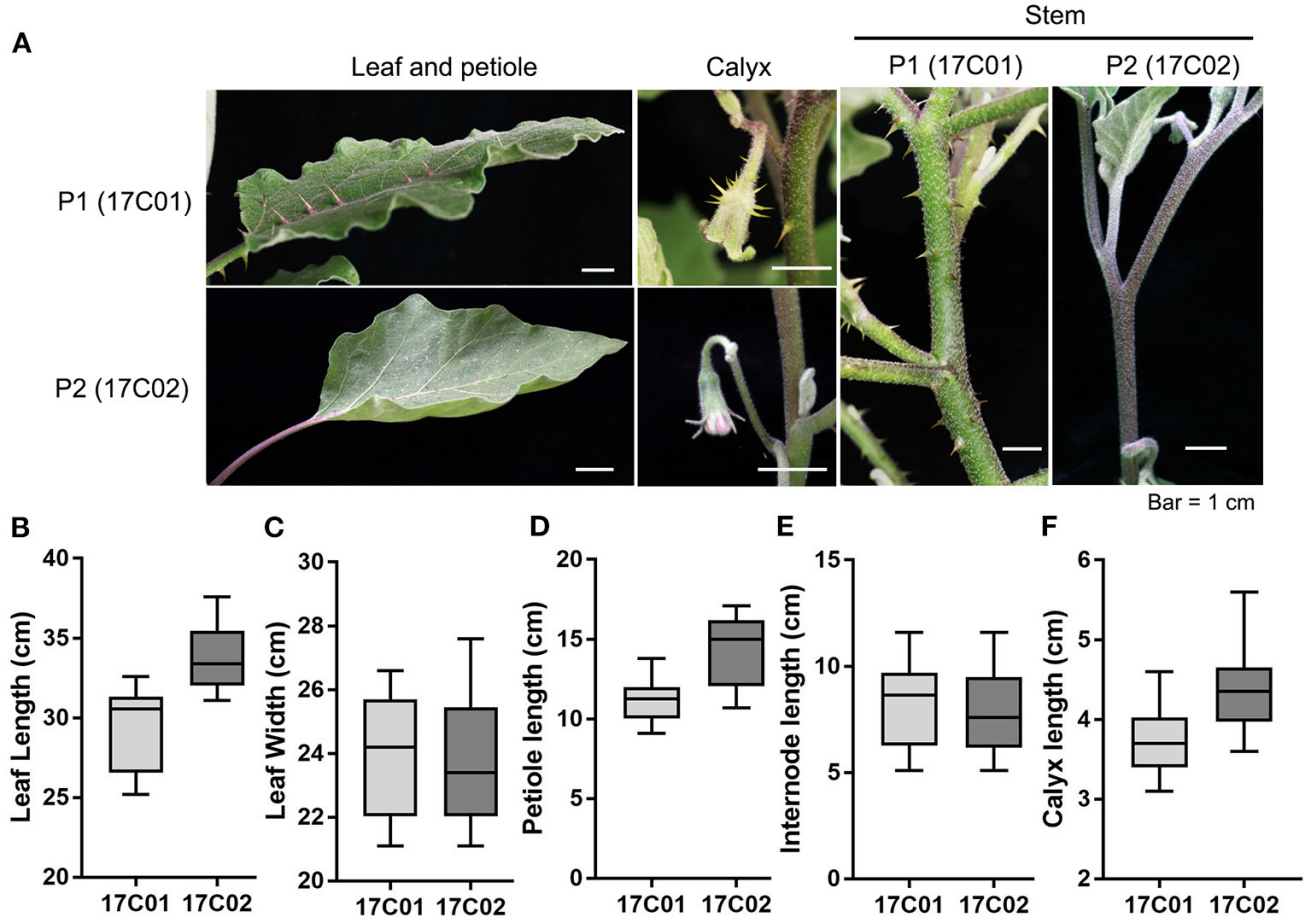
The prickle characteristics of 17C01 and 17C02 on stem, leaf, petiole, and calyx. **(A)** The apparent prickles were observed on leaf, petiole, calyx, and stem in 17C01, while few could be observed in 17C02. The length and width of leaf, petiole length, internode length, and calyx length of 17C01 and 17C02 are shown in **(B–F)**, respectively. At least 15 seedlings were selected to be measured at the fruiting period. The largest leaf was chosen to measure the leaf length, leaf width, and petiole length. The length of internode between the first and second branches and the calyx length 15 days after pollination were measured.

The cultivated eggplant “17C02” (*Solanum melongena* L.) and wild eggplant “17C01” (*Solanum insanum* (L.) Banfi, Galasso & Bartolucci) were used as male and female parents, respectively. The seeds used in this study were obtained from the seeds bank of the Beijing Vegetable Research Center. “17C02” is an inbred line with few prickles, whereas “17C01” is prickly (Figure 1; Supplementary Figure 1). An interspecific F2 population containing 308 individuals was generated from a cross between “17C01” and “17C02,” which was then used as the mapping population. BC1P1 (44 seedlings) and BC1P2 (44 seedlings) populations derived from F1 progeny backcrossed to P1 and P2, respectively, were also used for the genetic analysis. The parents and these populations were grown in the spring of 2018 in the greenhouses at the experimental field of the Beijing Vegetable Research Center, Beijing Academy of Agriculture and Forestry, Beijing, China, with a plant spacing of 50 cm, row spacing of 1.2 m, and ridge cultivation. Fertilization and irrigation followed the standard practice and were consistent for all experimental materials in the study.

### Investigation of Prickle Numbers

The eggplant materials, “17C01” (P1), “17C02” (P2), F1 (P1 × P2), F2 (*n* = 308), BC1P1 (*n* = 44), and BC1P2 (*n* = 44), were tested in the genetic analysis for four agronomic traits involved with prickles, including the number of prickles on the stem (PS), on the leaf (PL), on the petiole (PP), and on the calyx (PC). The prickles were visible 2 months after sowing, and the numbers of prickles on stems, petioles, calyxes, and leaves were recorded (Figure 1). For the two parents, we investigated 10 plants per parent for the 4 prickle traits. For each plant, three stems, leaves, and fruits were investigated, and the average phenotype value of the 10 plants of the parent was used as the trait value. For BC and F2 population, the prickle numbers that were recorded for each individual with at least 10 leaves, petioles, and calyxes were examined for trait investigation. The phenotypic data were analyzed using SPSS Statistics version 19.0 (SPSS Inc., IBM Corp., Chicago, IL, USA). Significance was taken at *P* < 0.05.

### The Re-sequencing of F2 Progeny

The re-sequencing was performed by Biomarker Technology Co., Ltd. (http://www.biomarker.com.cn/). A total of 100 randomly selected F2 individuals and both parents were used for the DNA re-sequencing analysis. The DNA was extracted from fresh leaves using the cetyltrimethylammonium bromide (CTAB) procedure (Chen and Ronald, 1999). The DNA sequencing was performed on the Illumina HiSeq2500 (Illumina, Inc., San Diego, CA, USA). The low-quality SNPs were filtered following these standards: (1) removed reads with adapters, (2) if the proportion of *n* (i.e., the specific base type cannot be determined) on a read was greater than 10%, the pair-end reads will be filtered out, and (3) removed low-quality reads (i.e., the number of bases with a quality value (*Q* ≤ 10) accounts for more than 50% of the entire reads). The filtering script is a Perl script written by a sequencing company. The clean reads were aligned to the *S*. *melongena* reference genome (i.e., Eggplant genome consortium V3) (https://solgenomics.net/organism/Solanum_melongena/genome) assembly by using the Burrows-Wheeler Aligner (BWA) software (Li et al., 2009). The SNP calling was performed by the Genome Analysis Toolkit (GATK) software. According to the localization of clean reads in the reference genome, the Picard (http://sourceforge.net/projects/picard/) was used to mark duplicates. The GATK software version 3.6 was used for local realignment and base recalibration (Li and Durbin, 2009; McKenna et al., 2010). These methods ensured the accuracy of SNPs. Finally, we used GATK to detect and filter SNP and then to obtain SNP datasets. Polymorphic SNPs between the parents were used for bin calling. Genotypes of the F2 population were determined based on the SNP positions. Only genotype aa × bb was further used in this study (Hu et al., 2018).

### Genetic Linkage Map Construction

The genetic linkage map was constructed by Biomarker Technology Co., Ltd. (http://www.biomarker.com.cn/). Bin markers were divided according to the recombination of offspring. The SNP between the recombination breakpoints is classified as a Bin. It is considered that there is no recombination event in Bin. Finally, the genetic map was constructed with Bin as a mapping marker. Bin with a length <10 kb or with severe partial segregation (*P* < 0.001) was screened out. The genotypes were compared across a 3-kb interval, and adjacent intervals with identical genotypes across all F2 individuals were combined into a recombination Bin (Block). The linkage map was constructed using the recombination Bin markers *via* HighMap software (Liu et al., 2014). When a Bin marker exhibited a high logarithm of odds (LOD) score, while the LOD values of its flanking Bin markers were zero, the marker was considered as a false one. Markers were assigned into linkage groups (LGs) based on their positions in the eggplant genome. The LOD scores between markers were used to confirm the effectiveness of markers for each LG. The Kosambi mapping function was used to estimate the genetic distance (cM) of adjacent markers.

### QTL Analysis

The QTL analysis was performed by Biomarker Technology Co., Ltd. (http://www.biomarker.com.cn/). The QTL analysis was carried out using the R/qtl in R software version 3.2.3. QTLs were called using the forward and backward regression method at the LOD threshold of 2.5 based on 3,000 replications at α = 0.05 and a walk speed of 1.0 cM. The QTLs were named based on the following pattern: *q* plus trait abbreviation, followed by the chromosome number (Chr.) of the QTL. The determination coefficient (*R*^2^, %) was calculated to indicate the percentage of the phenotypic variance explained by the effects of a specific QTL.

### DNA and RNA Extraction and the Cloning of Candidate Genes

Genomic DNA was isolated from fresh leaves of plants from the F2 mapping population and two parents using the CTAB procedure (Chen and Ronald, 1999). Total RNA was extracted from the leaf samples using a plant RNeasy kit (Tiangen, Beijing, China). RNA samples were reverse-transcribed into cDNA with a PrimeScript™ RT reagent Kit (Takara, Osaka, Japan). The cloning primers were designed based on the eggplant genome sequences (https://solgenomics.net/organism/Solanum_melongena/genome) (Barchi et al., 2019). The genomic sequences and the coding sequence (CDS) were then amplified from the genomic DNA and cDNA, respectively.

### Quantitative Real-Time PCR

Real-time PCRs were performed using the SYBR Green I Master Mix and were quantified with a Light Cycler 480 II instrument (Roche, Basel, Switzerland). The PCR program comprised an initial step at 94°C for 30 s, followed by 40 cycles of 94°C for 10 s and 58°C for 30 s. The amplification was followed by heating for 1 min at 60–95°C for the melting curve analysis. Each sample reaction was performed with three replications using 5 μl of Master Mix, 0.25 μM of each primer, 1 μl of diluted cDNA, and DNase-free water to a final volume of 10 μl. The lettuce EF1α genes were used as internal controls to normalize the transcript levels of target genes following the study by Pang et al. (2017). Relative gene quantification was calculated by the comparative ΔΔCT method (Schmittgen and Livak, 2008). The average 2^−ΔΔCT^ values were used to determine the differences in gene transcript levels. The lengths of PCR products were between 300 and 500 bp. The PCR products were sequenced to confirm the gene-specific amplification. The primers were designed using the Primer Premier 6.0 software and are shown in Supplementary Table 11.

### Phylogenetic Analysis

The full length of WUSCHEL amino acid sequences in eggplant, *Arabidopsis*, maize, and rice was downloaded from Eggplant genome consortium V3 (https://solgenomics.net/organism/Solanum_melongena/genome), TAIR (https://www.arabidopsis.org), MaizeGDB (https://www.maizegdb.org), and the MSU Rice Genome Annotation Project Database (http://rice.plantbiology.msu.edu/index.shtml), respectively, and were used in phylogenetic analysis. The ClustalW method embedded in MEGA 7.0 software was used for sequence alignment (Kumar et al., 2016). Then, the alignment file was subjected to construct the Neighbor-Jointing tree (Bootstrap = 1,000) using the Poisson model in MEGA 7.0 software (Kumar et al., 2016).

## Results

### Phenotypic Characterization of Prickles in Eggplant

**Figure 2:**
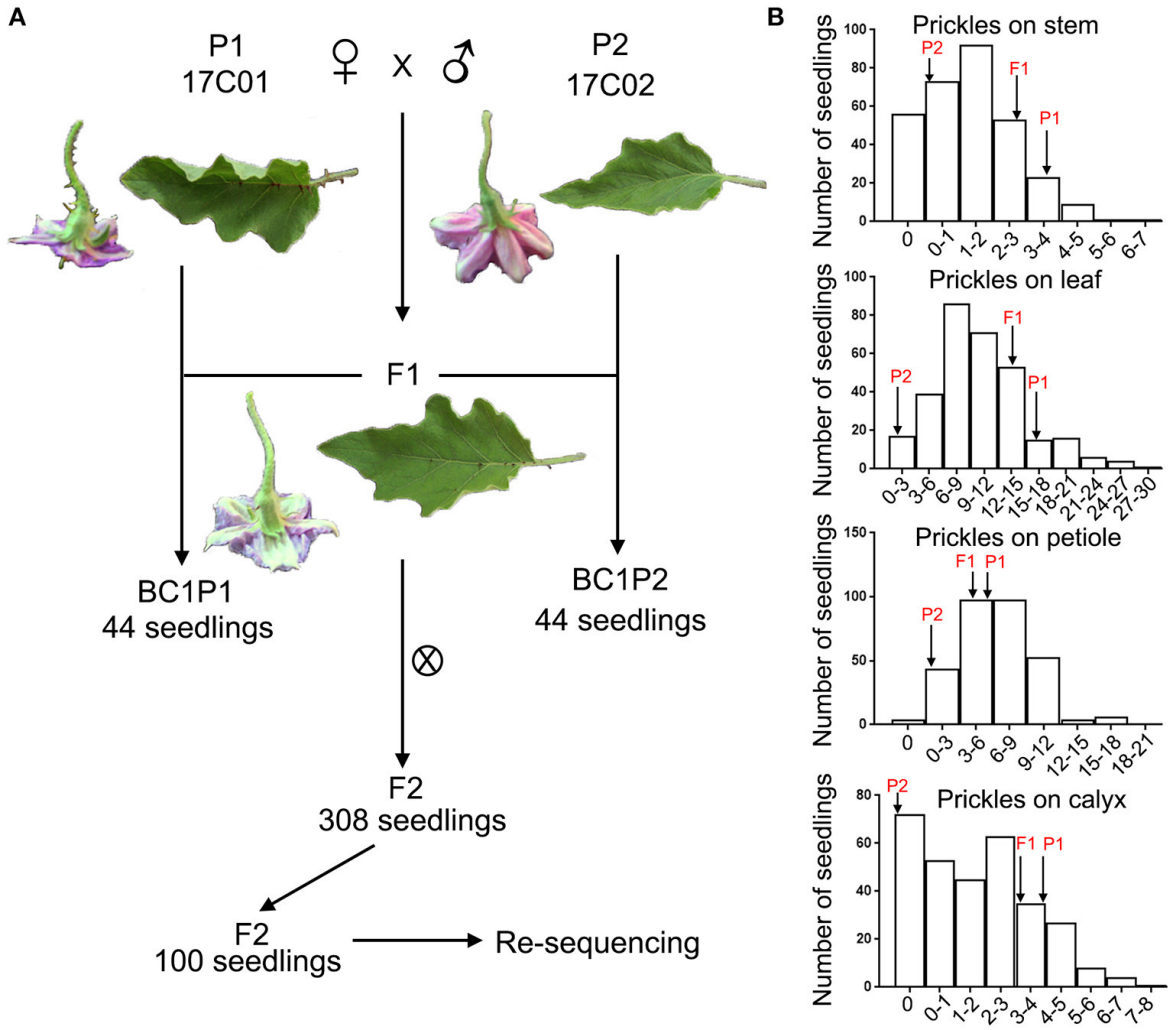
The construction of segregating populations and the distribution of prickles on stem, leaf, petiole, and calyx in these populations. **(A)** The construction of F1, F2, BC1P1, and BC1P2 populations. **(B)** Frequency distribution of the numbers of prickles on the stem, leaf, petiole, and calyx in the F2 population. Arrows indicate the prickle numbers of two parents, 17C01 and 17C02, and the F1 population.

To investigate the QTL controlling the development of prickles in eggplant, we constructed the segregating populations derived from crossing 17C01 and 17C02. 17C01, as the female parent (P1), is a wild landrace with prickles on the stem, petiole, leaf, and calyx, while 17C02, the male parent (P2), is almost prickless (Figure 1A). The size of organs bearing prickles was also measured (Figures 1B–F). The prickle numbers on leaf, petiole, and calyx in 17C01 are more than those in 17C02, while these organs are smaller in 17C01 than those in 17C02 (Figures 1B–F and Supplementary Table 1). The numbers of prickles on calyx of F2 population individuals were distinct and are shown in Supplementary Figure 1. The F1 seedlings exhibited prickly stem, petiole, leaf, and calyx such as 17C01, indicating that the prickly allele is likely dominant to non-prickly allele. BC1P1 and BC1P2 populations, both containing 44 individuals, were derived from the backcross of F1 to P1 or P2. The F2 population contained 308 seedlings (Figure 2A).

The numbers of prickles on stem, petiole, leaf, and calyx were examined in the segregating populations (Figure 2B). The average numbers of the prickles on the stem of the 17C01 population were 3.6, 16.4 on the leaf, 5.3 on the petiole, and 4.9 on the calyx, while these were 0.0, 1.1, 0.3, and 0.0 on average in the 17C02 population. The average prickle numbers of F1 were closer to those of 17C01 on all four tissues than to those of 17C02. The distribution of the number of prickles on stem, petiole, leaf, and calyx in the F2 population was continuous, indicating that the numbers of prickles on stem, leaf, petiole, and calyx are quantitatively inherited (Figure 2B and Supplementary Table 1).

### Inheritance of the Numbers of Prickles in Eggplant Populations

To clarify the inheritance of the prickles in eggplants, we performed the quantitative genetic analysis of the number of prickles on calyx, stem, leaf, and petiole using six generations, namely, P1, P2, F1, BC1P1 (BC1), BC1P2 (BC2), and F2. Following the mixed major-gene plus polygenic inheritance model, the Akaike's information criterion (AIC) values of 24 genetic models were obtained, among which three models with the lowest AIC value were selected for the suitability analysis (Supplementary Table 2). Among the three selected models, the 2MG-ADI model (i.e., two pairs of major gene-controlled additive-dominant-epistatic models), which had the smallest AIC value and contained fewer values with a significant difference, was considered as the optimal genetic model for the prickle numbers on the calyx. Thus, the prickle numbers on calyx were likely controlled by two pairs of major genes with additive, dominant, and epistatic effects in eggplant (Supplementary Table 3). 2MG-ADI, MX1-NCD-AD (i.e., a pair of negatively completely dominant major gene-controlled additive-dominant polygene model), and PG-ADI (i.e., polygene-controlled additive-dominant-epistatic genetic model) were determined as the optimal genetic models for prickle numbers on stem, leaf, and petiole, respectively (Supplementary Table 3). Furthermore, the values of the first-order and second-order genetic parameters of the optimal genetic model were calculated by the least square method, including the effect values and heritability of the major gene or polygenes (Supplementary Table 4). The first-order genetic parameter values suggested that the first pair of major genes mainly contribute to the dominant effect and that the additive effect of the two pairs of major genes (d_a_ > d_b_, |h_a_| > |h_b_|) controlled the prickle numbers on the calyx. The additive effect and dominant effect of the two pairs of major genes controlling the prickle numbers on the stem were similar (Supplementary Table 4). The second-order genetic parameter values showed that the traits of prickle number on the calyx and the stem were mainly inherited by the inheritance of major gene effect. The major gene heritability (${\text{h}}_{\text{mg}}^{2}$) values of BC1, BC2, and F2 generations were all higher than 50%, especially ${\text{h}}_{\text{mg}}^{2}$ of the prickle number on the calyx in the F2 generation was 79.7% (Supplementary Table 4). According to the heritability, the trait of prickle number on the leaf was mainly inherited by the inheritance of major gene effect in BC1 and F2 generations, while it was inherited mainly by the inheritance of polygene effect in BC2 generations (Supplementary Table 4). In addition, the trait of prickle number on petiole was mainly inherited by the inheritance of polygene effect, and the polygene heritability (${\text{h}}_{\text{pg}}^{2}$) of the F2 generation was 81.2% (Supplementary Table 4). These results indicated that the prickle numbers on different tissues are inherited through different genetic pathways in eggplant.

### Whole Genome Re-sequencing the F2 Populations

We selected 100 F2 individuals and two parents for the whole genome re-sequencing analysis on an Illumina HiSeq platform. A total of 18.95 and 17.14 million clean reads, containing 28.39 and 25.67 Gb data, were generated from the P1 and P2 samples, respectively (Supplementary Table 5). The average depths of sequencing for two parents were close to 21×. The base content, reads average error rate, and genome-wide distribution of read coverage of two parents are shown in Supplementary Figure 2. Meanwhile, the clean data of 151.78 Gb were obtained from the 100 F2 individuals with 92.75% of the average Q30 values (Supplementary Table 5). All the reads of P1, P2, and F2 populations were aligned to the eggplant reference genome (Barchi et al., 2019). An average of 96.08% clean reads from F2 individuals was mapped to the reference genome (Supplementary Table 5). There were 2,989,312 and 1,369,910 SNP markers being detected in two parents, P1 and P2 (Supplementary Table 6). Based on the variations in the sequences, an average of 458,370 high-quality SNPs was discovered from the F2 population (Supplementary Table 6).

### Construction of the High-Density Bin Genetic Linkage Map

**Figure 3:**
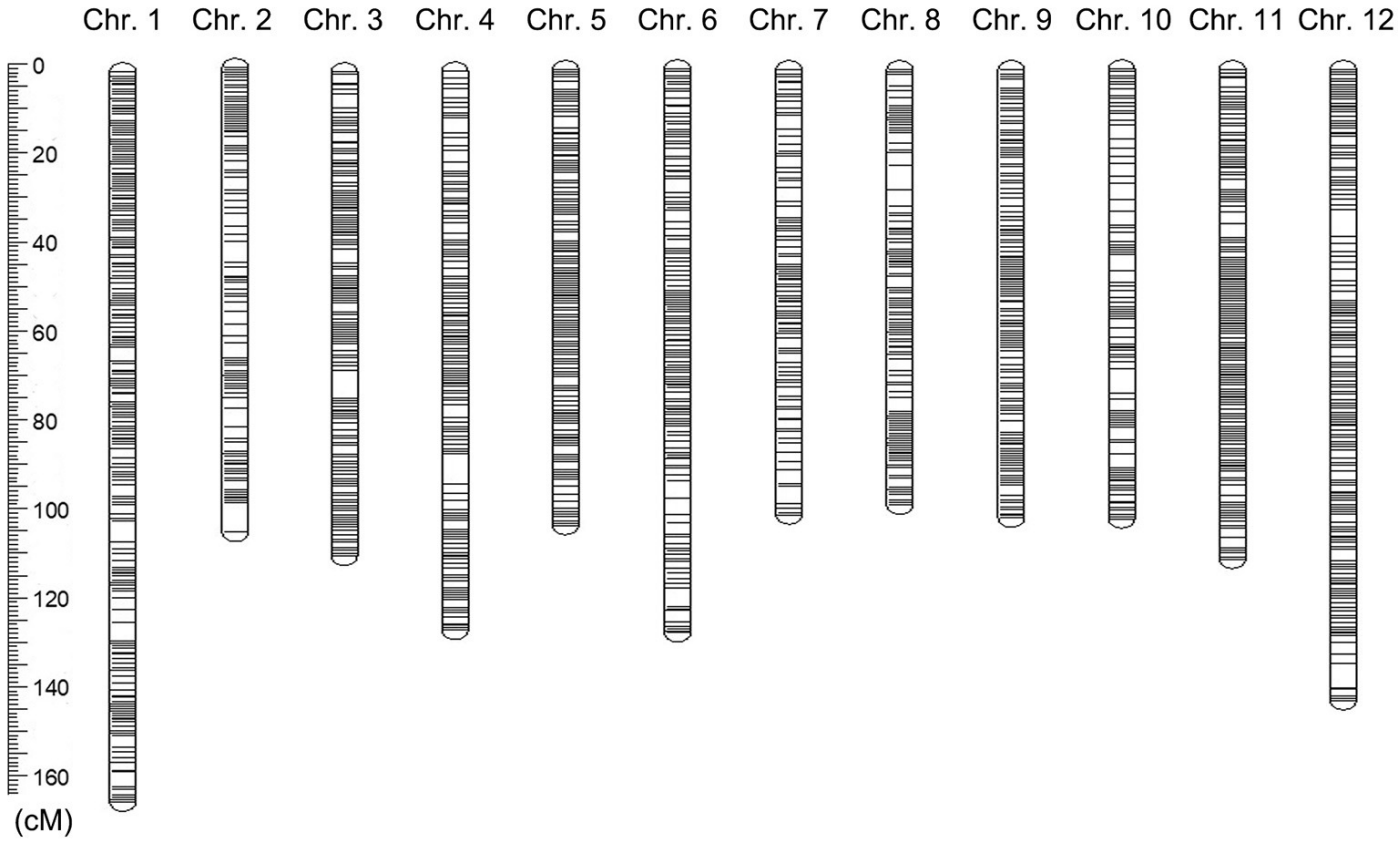
A graphic view of the distribution of markers on chromosomes in eggplant. The rounded rectangles represented the chromosomes in eggplant. The location and thickness of the black lines on the rounded rectangles indicating the location and distribution density of Bin markers on the chromosomes.

A group of markers was mapped to the same location formed a bin or a block. We used the sliding window method (i.e., 15 SNPs for each window sliding a site) to obtain the genotype and recombination sites and the Bin genotypes of the F2 population. Consequently, a total of 1,883,288 SNPs were developed into 278,891 Bin markers on 12 chromosomes (Supplementary Table 7). According to the recombination in the F2 population, A total of 3,918 recombination Bins were used to construct the high-density Bin genetic map (Supplementary Table 8 and Supplementary Figure 3). On the 12 chromosomes, these Bins spanned 1,384.62 cM with a range of 97.93 cM (chromosome 8) to 164.21 cM (chromosome 1) and with a density of 0.58 cM between adjacent bin markers (Supplementary Table 8). There was an average density of 0.42 cM, from 0.26 to 0.64 cM between adjacent Bin markers for various chromosomes (Supplementary Table 8 and Figure 3).

To clarify the pattern of recombination and exchange in the F2 population, we performed the graphical genotype analysis of F2 progeny using 3,918 Bin markers. Some F2 individuals contained a few non-recombined chromosomes in their genomes, implying that they were from a single parent genome (Supplementary Figure 4A). To understand the linear and genomic variation, we compared the genetic map and reference genome by constructing the linear relationship diagrams between the genetic and physical maps (Supplementary Figure 4B). The Spearman's correlation coefficients were from 0.84 to 0.99 with an average value of 0.96. These results suggested a high-quality genetic linkage map constructed using the re-sequencing data of the F2 population.

### QTL for the Number of Prickles in Stem, Leaf, Petiole, and Calyx

**Figure 4:**
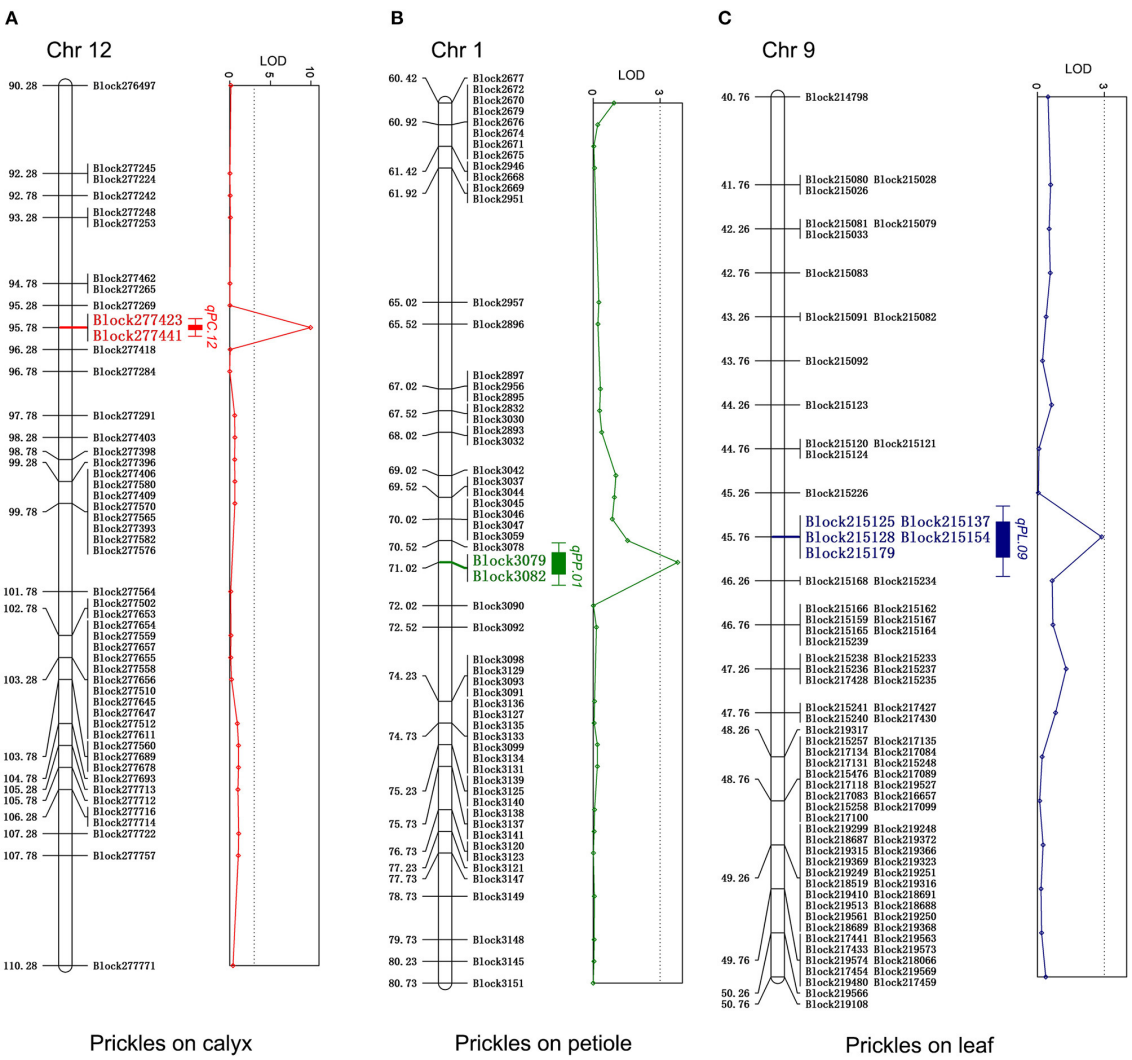
Quantitative trait locus (QTL) regions associated with the number of prickles on calyx **(A)**, petiole **(B)**, and leaf **(C)** with the logarithm of odds (LOD) values of bin markers. The markers within QTL regions associated with the prickles on calyxes, petiole, and leaf were highlighted with red, green, and blue, respectively. The diagrams of LOD values were represented on the right.

For the four prickle traits, there are eight QTLs distributed in eight chromosomes, among which five QTLs were detected as controlling the numbers of prickles on the stem, while the numbers of prickles on the calyx, leaf, and petiole were controlled by a single QTL, respectively (Table 1, Figure 4, and Supplementary Figure 5). Based on the chromosomes that these QTLs were located, the QTLs conferring the numbers of prickles on the stem, calyx, leaf, and petiole were designated as *qPS.03, qPS.04, qPS.05, qPS.06, qPS.08, qPC.12, qPL.09*, and *qPP.01*, respectively (Table 1). A major QTL, *qPC.12*, was identified near Block277423 on chromosome 12. The LOD value of *qPC.12* was high up to 9.96, while the LOD value of *qPL.09* and *qPP.01* was 2.88 and 3.78, respectively (Table 1 and Figure 4). The phenotypic variation explained (PVE) value of *qPC.12* and *qPL.09* was 30.42% and 13.26%, respectively, while the PVE of *qPP.01* was only 2.96% (Table 1 and Figure 4). On chromosomes 3, 4, 5, 6, and 8, five QTLs were detected as controlling the numbers of prickles on the stem with the PVE of 0.97–11.73% and the LOD values of 2.07–2.4, of which two major QTLs, *qPS.05* and *qPS.06*, could account for 10.03% and 11.73% of the PVE, respectively (Table 1 and Supplementary Figure 5). The LOD values of the whole genome are listed in Supplementary Table 9.

**Table 1:** Quantitative trait loci (QTLs) for the prickle numbers on calyx, stem, leaf, and petiole.

**Trait**	**QTL name**	**Chr**	**Franking markers**	**LOD**	**Additive**	**Dominant**	**PVE (%)**
Prickles on calyx	*qPC.12*	12	Block277423–Block277441	9.96	1.40	0.51	30.42
Prickles on leaf	*qPL.09*	9	Block215137–Block215154	2.88	2.00	−2.72	13.26
Prickles on petiole	*qPP.01*	1	Block3079–Block3082	3.78	−0.77	0.68	2.96
Prickles on stem	*qPS.03*	3	Block100758–Block100829	2.40	0.01	0.26	0.97
	*qPS.04*	4	Block127179–Block127199	2.16	−0.02	0.68	6.68
	*qPS.05*	5	Block130102–Block130194	2.24	0.57	0.15	10.03
	*qPS.06*	6	Block172319–Block172338	2.07	0.24	−0.83	11.73
	*qPS.08*	8	Block211580–Block211741	2.19	0.32	0.20	3.46

*LOD, logarithm of odds; PVE, phenotypic variation explained*.

Among the three single QTLs conferring the numbers of prickles on the calyx, leaf, and petiole, *qPC.12* has the highest LOD value of 9.96 and the PVE value of 30.42%. The region from Block277423 to Block277441 spanned about 7 kb on chromosome 12 in *qPC.12* (Table 1 and Supplementary Table 10). In this region, there were seven putative genes, with one gene being uncharacterized (Table 2). The two adjacent genes, *SMEL_012g394430* and *SMEL_012g394440*, encode WUSCHEL-related homeobox 3-like proteins with the same homologous gene in *A. thaliana, AT2G28610*. Another two contiguous genes, *SMEL_012g394400* and *SMEL_012g394410*, encode the proteins belonging to chitinase (Table 2). The coding proteins of two other genes, *SMEL_012g394380* and *SMEL_012g394390*, are the putative homologs of DNA-directed RNA polymerase III subunit rpc1-like isoform X1 and DNA ligase 1-like, respectively (Table 2).

**Table 2:** Genes in the region of the QTL for the prickle numbers on the calyx.

**Gene ID**	**Chr**	**Start (bp)**	**Stop (bp)**	**Arabidopsis homolog**	**Annotation**
SMEL_012g394380	12	92858720	92891580	AT5G60040	DNA-directed RNA polymerase III subunit rpc1-like isoform X1
SMEL_012g394390	12	92893127	92893897	AT5G60030	DNA ligase 1-like
SMEL_012g394400	12	92896532	92897452	AT1G14500	Chitinase 1-like
SMEL_012g394410	12	92898771	92899691	AT5G23390	Chitinase 2-like
SMEL_012g394420	12	92902268	92903728	AT2G41475	Uncharacterized protein LOC104644786
SMEL_012g394430	12	92919594	92921639	AT2G28610	WUSCHEL-related homeobox 3-like
SMEL_012g394440	12	92944508	92947244	AT2G28610	WUSCHEL-related homeobox 3-like

### Identifying Candidate Genes Involved in the Development of Prickle in the Calyx

**Figure 5:**
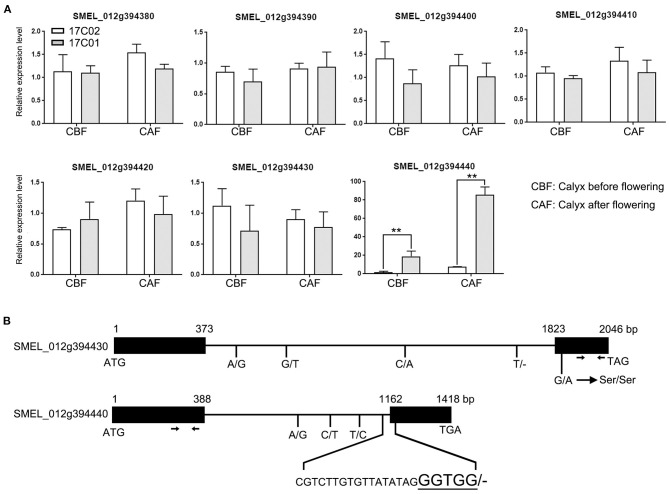
The expression analysis and sequence varieties of candidate genes related to the development of prickle in eggplant. **(A)** The expression profiles of seven candidate genes in the region of *qPC.12* in calyx before and after flowering in 17C01 and 17C02. The expression level of one of the three biological replications in 17C02 calyx before flowering was defined as “1.” Values represent means ± SD (*n* = 3) from three biological replicates. Statistical significance between the gene expressions in 17C01 calyx and 17C02 calyx before or after flowering was performed by a *t*-test: **P < 0.01. **(B)** The genomic sequence varieties in *SMEL_0112g394430* and *SMEL_0112g394440* between 17C01 and 17C02. The black rectangles and lines were designated as exons and introns, respectively. The nucleotide numbers from ATG to stop codon, TGA or TAG, were marked on the top of rectangles in each gene. The single nucleotide variations and deletion/insertion variations, in which the nucleotides in 17C01 and 17C02 were placed on the left and right of the slash, respectively, were written below. The short bar represented that there was no nucleotide in this site. The four magnified characters were designated as the nucleotides in the predicted exon. The primers of *SMEL_0112g394430* and *SMEL_0112g394440* used in the real-time quantitative reverse transcription PCR (qRT-PCR) were marked with arrows.

To further identify the putative functional genes determining the development of prickles on calyx in eggplant, we first compared the expression levels of the seven candidate genes in the calyx. We collected the calyx of 17C01 and 17C02 before flowering, when the petals were still fully in the calyx, and after flowering. The results of the real-time PCR showed that the expression level of *SEML_012g394440* was upregulated by nearly 20-fold and more than 80-fold in 17C01 calyx before flowering and after flowering, respectively, comparing with that in 17C02. The expression of other genes had no significant differentiation in the calyx of 17C01 and 17C02 (Figure 5A). The expression levels of *SEML_012g394440* in F2 individuals were also detected, and the prickly F2 individuals showed significantly higher expression levels of *SEML_012g394440* than those in the prickless lines (Supplementary Figure 7). The results suggested that the higher expression level of *SEML_012g394440* in 17C01 calyx probably conferred the formation of prickles on calyx in eggplant.

Both *SEML_012g394430* and *SEML_012g394440* encode WUSCHEL-related homeobox 3-like protein. To further confirm the calyx prickle-related functional gene, we examined the sequence variation of *SEML_012g394430* and *SEML_012g394440* between 17C01 and 17C02. The results showed that there were a total of five single nucleotide variations in *SEML_012g394430* between 17C01 and 17C02, among that four were in the intron region and one in the second exon leading to a synonymous mutation (Figure 5B and Supplementary Figure 8). In *SEML_012g394440*, three single nucleotide variations existed in intron region. Moreover, a 22-bp deletion, i.e., 4 bp in the second exon and 18 bp in the intron, was found in the 17C02 genome (Figure 5B and Supplementary Figure 8). This deletion was present in the conjunction region between intron and exon, which could cause the different splicing patterns, resulting in alterations in its function. These results suggest that *SEML_012g394440* is likely the functional gene involved in the development of prickles on the calyx.

**Figure 6:**
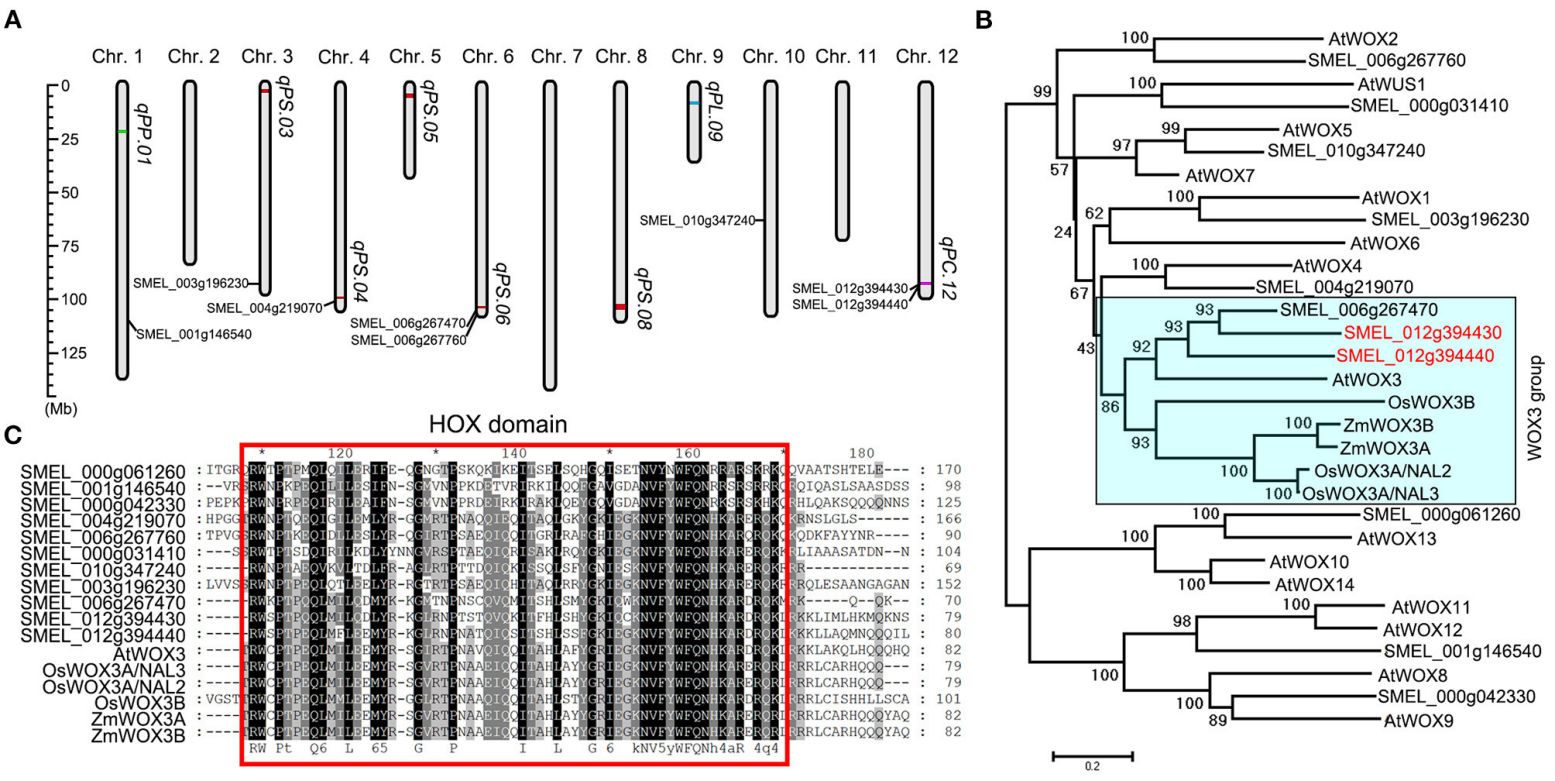
The genome-wide analysis of WUSCHEL gene family in eggplant. **(A)** The distribution of *SmWUS* genes and *qPS.03, qPS.04, qPS.05, qPS.06, qPS.08, qPC.12, qPL.09*, and *qPP.01* on chromosomes in eggplant. **(B)** The phylogenetic tree of *WUS* genes in rice, maize, eggplant, and *Arabidopsis thaliana*. The two eggplant *WUS* genes in *qPC.12* were represented in red. The clade containing *WOX3* proteins was in a blue box and designated as the *WOX3* group. **(C)** The alignment of members in the *WOX3* group and other *SmWUS* genes. Conserved HOX domains were highlighted with the red rectangle.

*SMEL_012g394440* gene belongs to the *WUSCHEL* (*WUS*) gene family that encodes the homeodomain transcription factor participating in regulating cell fate during cell dedifferentiation, including the size of shoot meristem, somatic embryo, shoot, and lateral leaf formation, by maintaining the pluripotent stem cells (Honda et al., 2018; Jha et al., 2020). We identified 11 *SmWUS* genes, with 8 located on 5 chromosomes in the eggplant genome, and 3 on unsigned contigs (designated as chromosome 0, Figure 6A). The phylogenetic analysis of *WUS* genes in rice, maize, *Arabidopsis*, and eggplant showed that *SMEL_012g394430* and *SMEL_012g394440* had a close phylogenetic relationship (Figure 6B). Both genes were clustered on the clade containing *AtWOX3, ZmWOX3A, ZmWOX3B, OsWOX3A*, and *OsWOX3B*, designated as the *WOX3* group (Figure 6B). The alignment of *SmWUS* proteins and *WOX3* in *Oryza sativa* (rice), *Zea mays* (maize), and *A. thaliana* indicated that all these proteins contained a conserved HOX domain in the N-terminal (Figure 6C and Supplementary Figure 9). These results suggest that *SMEL_012g394430* and *SMEL_012g394440* in *qPC.12* have a close evolutionary relationship with the trichome-formation-related gene *OsWOX3B*.

## Discussion

In this study, we identified the major QTLs conferring the numbers of prickles on the stem, leaf, petiole, and calyx in eggplant using a high-density genetic Bin map. Particularly, we identified the candidate functional gene *SMEL_0112g394430*, located in QTL *qPC.12*, encoding a WUSCHEL-related homeobox-like protein, involved in the development of prickles on the calyx. Our results represent significant progress on the characterization of the prickly phenotype in eggplant, which is beyond many previous studies that mainly focused on the identification of QTLs associated with the development of prickle, except the *Pl* gene identified in the study by Miyatake et al. (Doganlar et al., 2002; Frary et al., 2014; Gramazio et al., 2014; Portis et al., 2014, 2015; Miyatake et al., 2020). Our results benefited from the high-quality molecular markers based on the high throughput sequencing technology and reliable phenotyping data. The sequencing data obtained from two parents and F2 individuals generated a total of 45,837,036 high-quality SNPs and 3,918 recombination Bin markers with a density of 0.91 cM between adjacent Bin markers. The genetic linkage map with the high-quality and high-density markers enabled the map-based cloning of prickle responsible for the genes with high accuracy. We also performed the genetic analysis of the emergence of prickle on the calyx (data not shown). Intriguingly, the major QTL conferring the emergence of prickle on the calyx was also located on the same region with *qPC.12*, which confirms that the *qPC.12* is most likely the QTL responsible for the development of prickle on the calyx in eggplant.

According to the genetic model, the optimal genetic model of prickles on calyx was 2MG-ADI, which was an additive-dominant-epistatic genetic model controlled by two major genes (additive effects: 1.8536 and 0.7019, respectively). We mapped a candidate gene (encoding WUSCHEL-related homeobox-like protein) in 12 chromosomes, verified it in the parent and F2, and showed that it was likely to be one of the two major genes. Several major QTLs associated with prickly phenotype in eggplant were identified in previous studies. A major QTL was mapped to chromosome 6 using an interspecific mapping population (Doganlar et al., 2002; Frary et al., 2014; Gramazio et al., 2014; Portis et al., 2014; Miyatake et al., 2020), but we did not find another related QTLs on other chromosomes, which was most likely due to the differences in the genetic mapping materials we used. At the same time, according to the genetic models, this may also complement the QTLs of other populations of prickles on the calyx (Miyatake et al., 2020). Several QTLs were mapped to chromosomes 7 and 8 and were considered possibly controlling the prickle strength (Portis et al., 2014). In this study, we categorized the prickly phenotype base on their growth organ type. One of the QTLs conferring the numbers of prickles on the stem, *qPS.06*, was also located on chromosome 6 reported in previous studies (Frary et al., 2014; Gramazio et al., 2014; Miyatake et al., 2020). The PVE of *qPS.06* (11.73%) was highest among the five QTLs related to the prickle on the stem (Table 1). The candidate genes conferring prickless were also located on chromosome 6, 7, and 11 in selective sweep (SS) regions by re-sequencing 23 accessions of *S*. *melongena* (Barchi et al., 2021). We have identified additional QTLs conferring prickly phenotype, probably because the female parent, a cultivar, exhibiting a certain degree of prickly on the stem, leaf, and petiole. We categorized the prickly phenotype base on their growth organ type, allowing the identification of QTLs related to the development of prickles on a specific growth organ type. Some studies show that prickles on different organs may comprise a set of closely linked loci or, more likely, they represent a single pleiotropic locus (Portis et al., 2015; Wei et al., 2020). In this study, four prickle traits are mapped to eight different QTLs in eight chromosomes. From the genetic analysis, these traits are the different genetic models except prickles on the stem and calyx (2MG-ADI). One parent “17C01” has more prickles on the calyx, stem, leaf, and petiole, but another parent “17C01” has no prickles on the calyx, and it has few prickles on the stem, leaf, and petiole. The abovementioned evidence indicates that the prickles of different organs may be inherited independently and controlled by multiple loci.

In the region of *qPC.12*, there were seven annotated genes. The analysis of previous literature suggested that a few of them is less likely to be involved directly in the development of prickles on the calyx in eggplant. *SMEL_012g394400* and *SMEL_012g394410* are chitinase genes, while the homologous gene of *SMEL_012g394380*, i.e., *AT5G60040*, encodes a subunit of RNA polymerase III and DNA ligase gene. Furthermore, *AT5G60030*, the homologous gene of *SMEL_012g394390*, has not been characterized. The homologous gene of *SMEL_012g394420* in *A. thaliana* was designated as *EMBRYO-SPECIFIC PROTEIN 3A/ATS3* and has a putative function in stomatal closure (Van Hove et al., 2015). It is therefore left *SMEL_012g394430* and *SMEL_012g394440* being the possible candidate gene in the development of prickles on the calyx. *SMEL_012g394430* and *SMEL_012g394440* are the homologous genes of *AT2G28610* in *A. thaliana*. It is known that *AT2G28610* encodes WUSCHEL-related homeobox 3/WOX3, one of the WUSCHEL proteins, and the gene is involved in regulating lateral axis-dependent development of flowers and cell proliferation in *A. thaliana* (Nakata et al., 2012; Cho et al., 2013; Niu et al., 2015; Zhang et al., 2019, 2020b). In *O. sativa* (rice), the functional gene conferring the formation of trichome was reported to also encode a WUSCHEL-related homeobox 3B protein and was identified as *depilous* (*dep*) in glabrous chromosome segment substitution line (GLSL15) (Angeles-Shim et al., 2012), *NUDA*/*GL1* in the glabrous Yunnan upland Nuda rice (Zhang et al., 2012), and *Glabrous Rice 1* (*GLR1*) in a glabrous variety Jia64 (Li et al., 2012). The molecular mechanism of *OsWOX3B* in the growth regulation of trichome in rice was subsequently characterized. *OsWOX3B* could interact with an AP2/ERF transcription factor, HL6, which was also responsible for the development of trichome and functioned together to regulate rice macro-hair elongation likely involving the role of auxin (Sun et al., 2017). Future research is required to verify the functions of two *WOX3* genes, *SMEL_012g394430* and *SMEL_012g394440* in *qPC.12* in eggplant. Whether *SMEL_012g394430* and *SMEL_012g394440* could interact with HL6 to regulate the growth of prickle demands further investigations.

It is worth noting that we found a total of 11 WUSCHEL genes in the eggplant genome locating at 6 of the 12 chromosomes and that they all contained the conserved HOX protein domain (Figures 6A,C and Supplementary Figure 9). Previous studies showed that the HOX protein domain in the WUSCHEL-related homeobox 3B protein is important for the development of trichome in rice (Angeles-Shim et al., 2012; Li et al., 2012; Zhang et al., 2012). Our phylogenetic analysis revealed another *WUSCHEL* gene, i.e., *SMEL_006g267470*, in the *WOX3* group, with a sequence similar to the two *WUSCHEL* genes in *qPC.12* (Figure 6B). *SMEL_006g267470* on chromosome 6 is near *qPS.06* in our study and also appears to be in the genomic region associated with prickly phenotype as reported in several studies. *SMEL_004g219070* was located in the genome near *qPS.04* (Figure 6A). Whether *SMEL_006g267470* and *SMEL_004g219070* play a role in the regulation of the development of prickle remains to be further investigated. Available studies suggested that the WUSCHEL gene family plays important roles in cell fate during cell dedifferentiation, including the size of shoot meristem, somatic embryo, adventitious shoot, and lateral leaf formation, by maintaining the pluripotent stem cells (Honda et al., 2018; Jha et al., 2020). The 11 *SmWUS* genes in eggplant as reported in our study probably participate in regulating plant growth and developmental processes. The functional characterization of these genes will enrich the understanding of the growth regulation mechanism in eggplant.

## Conclusion

In this study, we constructed a high-quality genetic linkage map using the populations derived from the cross of a prickly wild landrace 17C01 and a cultivated variety 17C02, by whole genome re-sequencing 100 F2 individuals. The major QTLs conferring the numbers of prickles on the stem, leaf, petiole, and calyx were identified. A candidate functional gene, encoding a WUSCHEL-related homeobox 3B protein, in *qPC.12* was proposed as the candidate gene controlling the development of prickle on the calyx. The results obtained from this study would ultimately facilitate uncovering the molecular regulatory mechanisms underlying the development of prickle in eggplant and inform regarding the marker-associated breeding of eggplant variety with desirable agronomic traits.

## Data Availability Statement

The datasets presented in this study can be found in online repositories. The names of the repository/repositories and accession number(s) can be found below: https://bigd.big.ac.cn/gsa (Wang et al., 2017; CNCB-NGDC Members and Partners, 2019), CRA004017.

## Author Contributions

DL, ZQ, and YC designed the experiments. ZQ and JZ constructed the F2 population and performed the phenotypic investigation. HC, LL, and MD carried out the experiment. BZ, ZQ, and JZ analyzed the data. BZ and DL wrote the paper. All authors contributed to the article and approved the submitted version.

## Conflict of Interest

The authors declare that the research was conducted in the absence of any commercial or financial relationships that could be construed as a potential conflict of interest.

## Publisher's Note

All claims expressed in this article are solely those of the authors and do not necessarily represent those of their affiliated organizations, or those of the publisher, the editors and the reviewers. Any product that may be evaluated in this article, or claim that may be made by its manufacturer, is not guaranteed or endorsed by the publisher.
