# Supplementary material for: Identification of Quantitative Trait Loci Controlling the Development of Prickles in Eggplant by Genome Re-sequencing Analysis
Source: Front Plant Sci. 2021 Sep 8;12:731079. doi: 10.3389/fpls.2021.731079 (PMC8457335; doi:10.3389/fpls.2021.731079)
Supplement: Supplementary file 2 [file Data_Sheet_1.PDF]

Supplementary Material

Identification of QTLs controlling the development of prickles in eggplant by genome re-sequencing analysis

Zongwei Qian*, Bin Zhang*

*** Correspondence:** Yanling Cui, cuiyanling@nercv.org
Dayong Li, lidayong@nercv.org, dyli2003@126.com

1 Supplementary Figures and Tables

1.1 Supplementary Figures

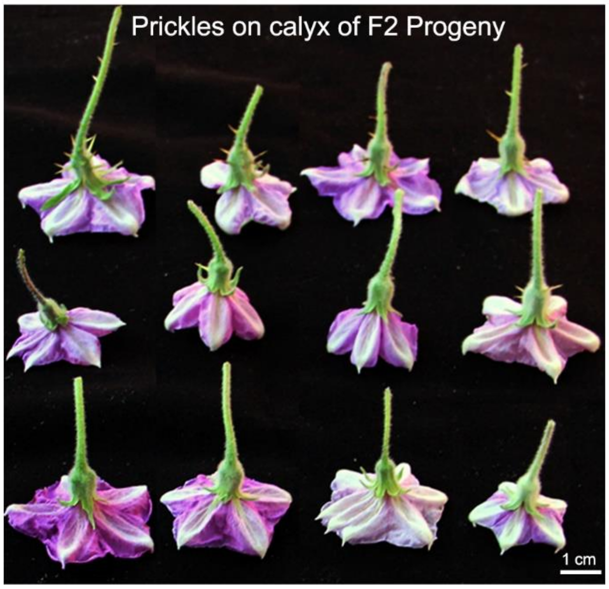

Supplementary Figure 1. The prickles on calyxes of F2 progeny derived from 17C01 and 17C02.

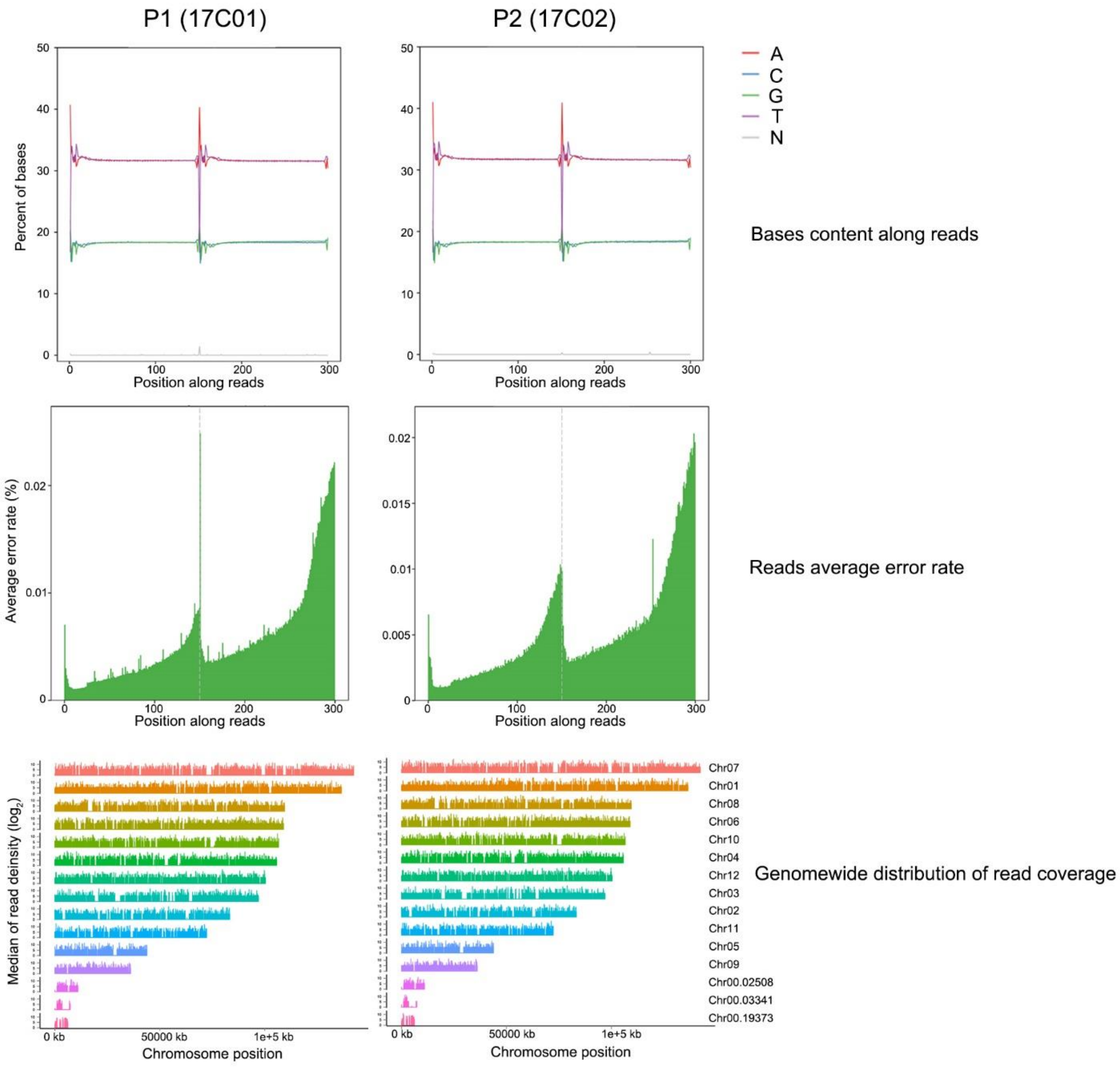

Supplementary Figure 2. The re-sequencing quality of 17C01 and 17C02.

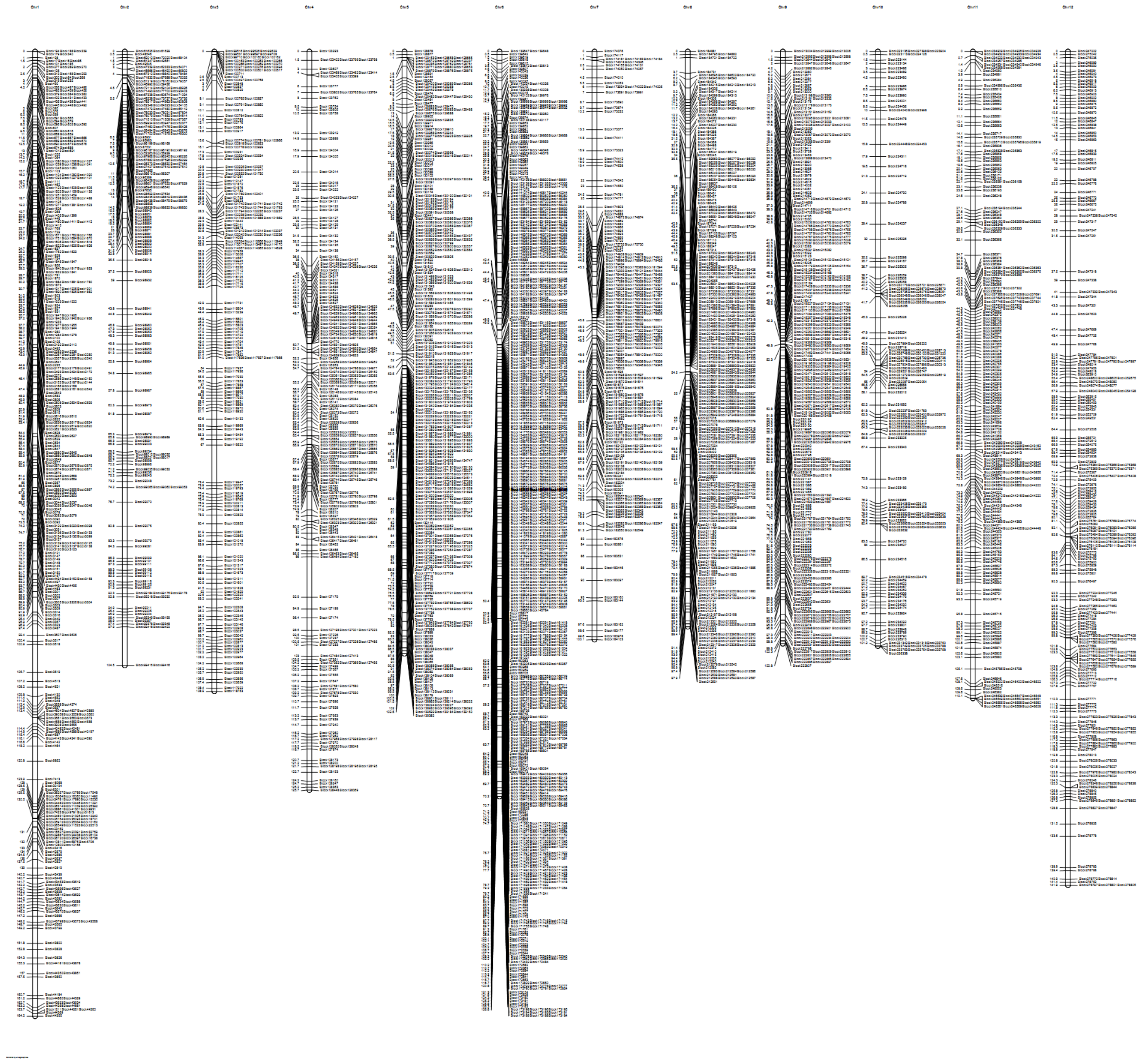

Supplementary Figure 3. The high-density genetic linkage Bin map. The Bin markers were named as Block and ranked based on the chromosome locations.

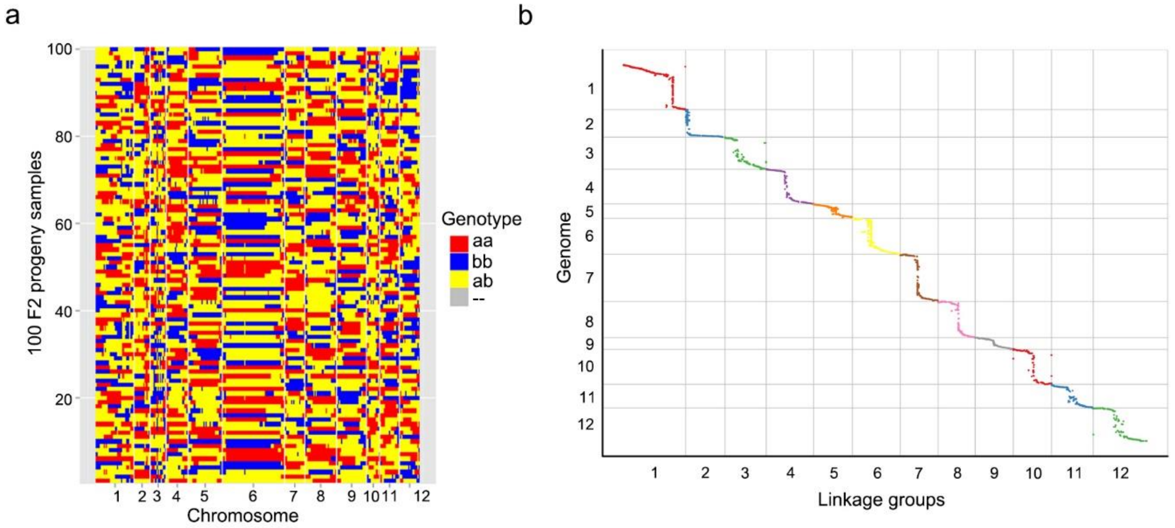

Supplementary Figure 4. The graphical genotype analysis of F2 progeny (a) and the linear relationship diagrams between the genetic and physical maps (b).

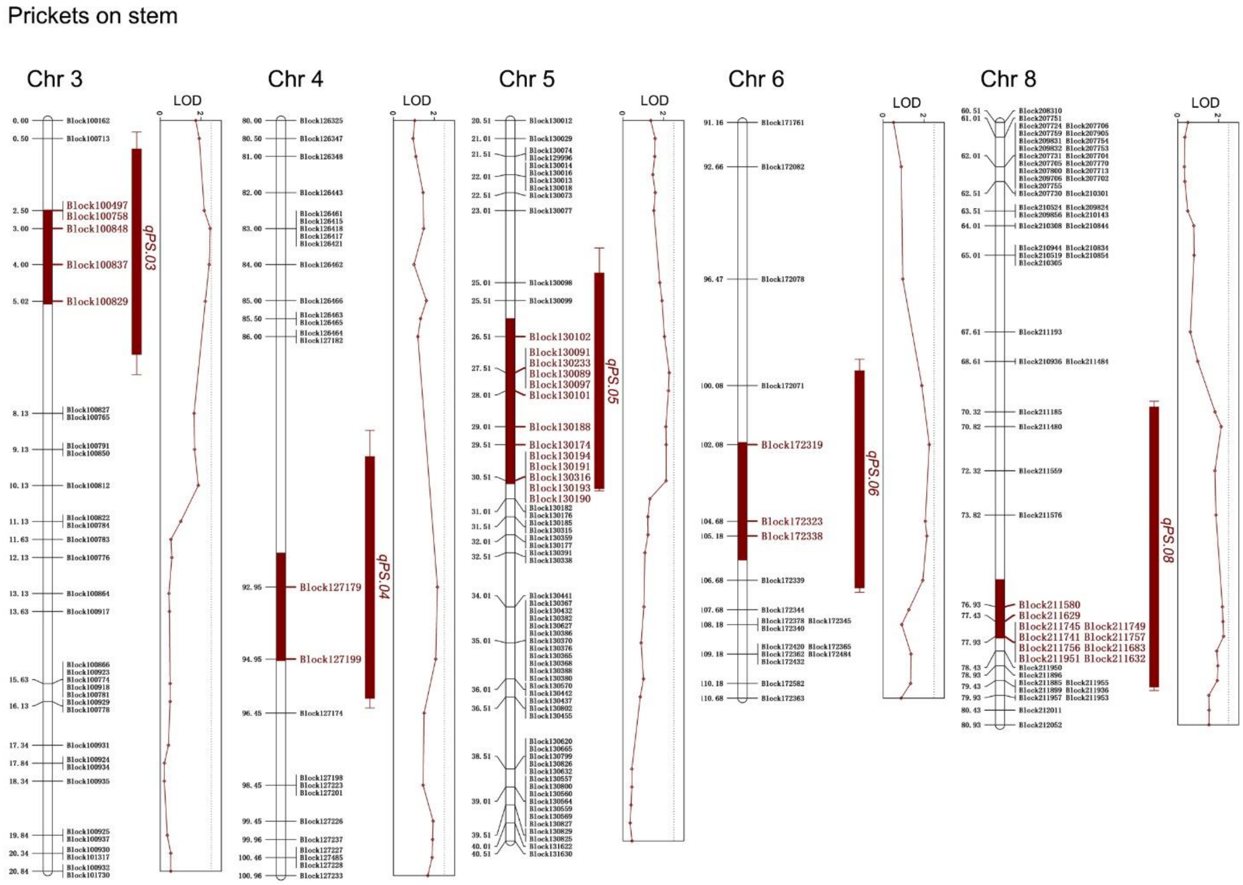

Supplementary Figure 5. QTL regions associated with the number of prickles on stem with the LOD values of Bin markers.

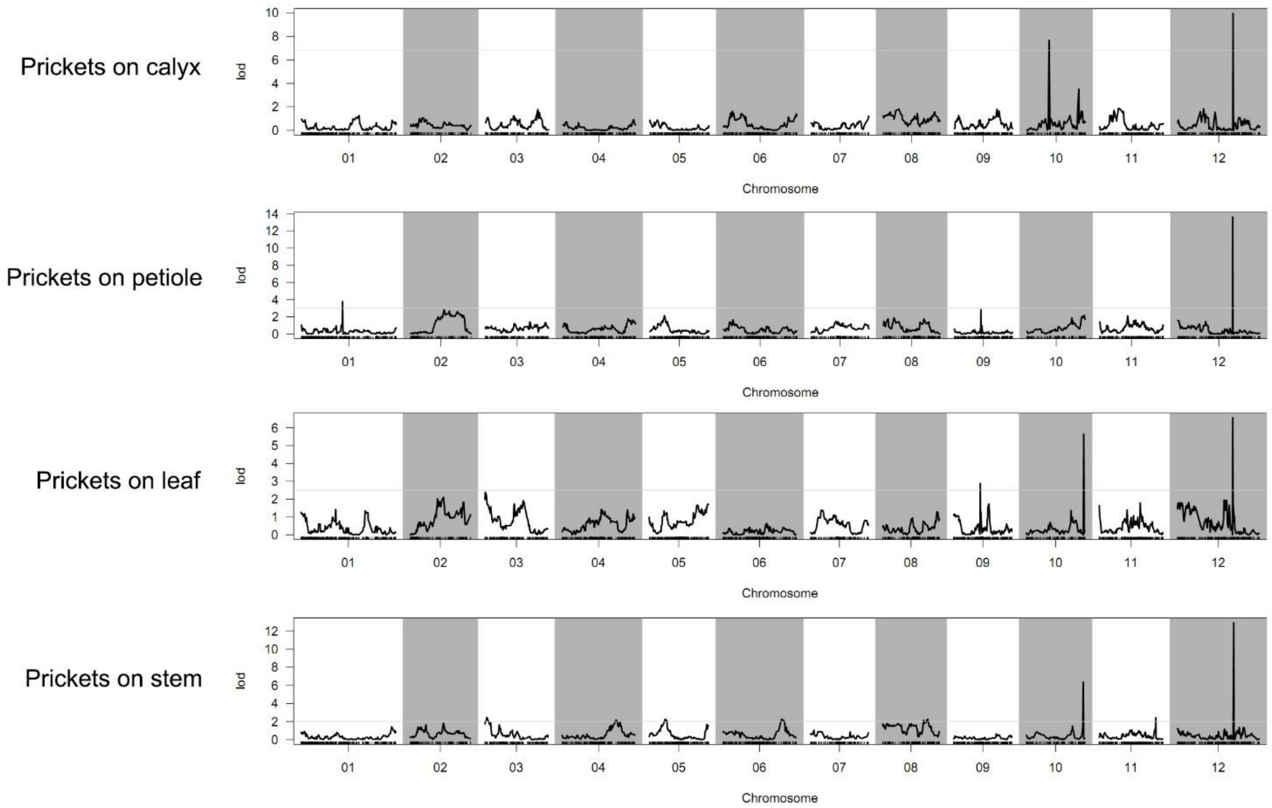

Supplementary Figure 6. The Bin marker LOD values diagrams for the number of prickles on stem, leaf, petiole and calyx in eggplant.

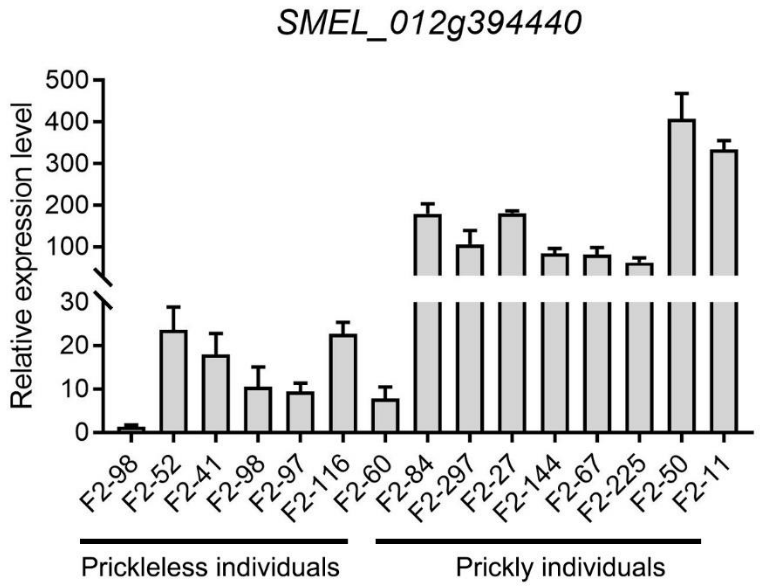

Supplementary Figure 7. The expression levels of *SMEL_012g394440* in F2 individuals. The expression level of *SMEL_012g394440* in F2-98 was designated as "1". Three calyxes were mixed in one sample and three biological replicates were collected in each F2 individual.

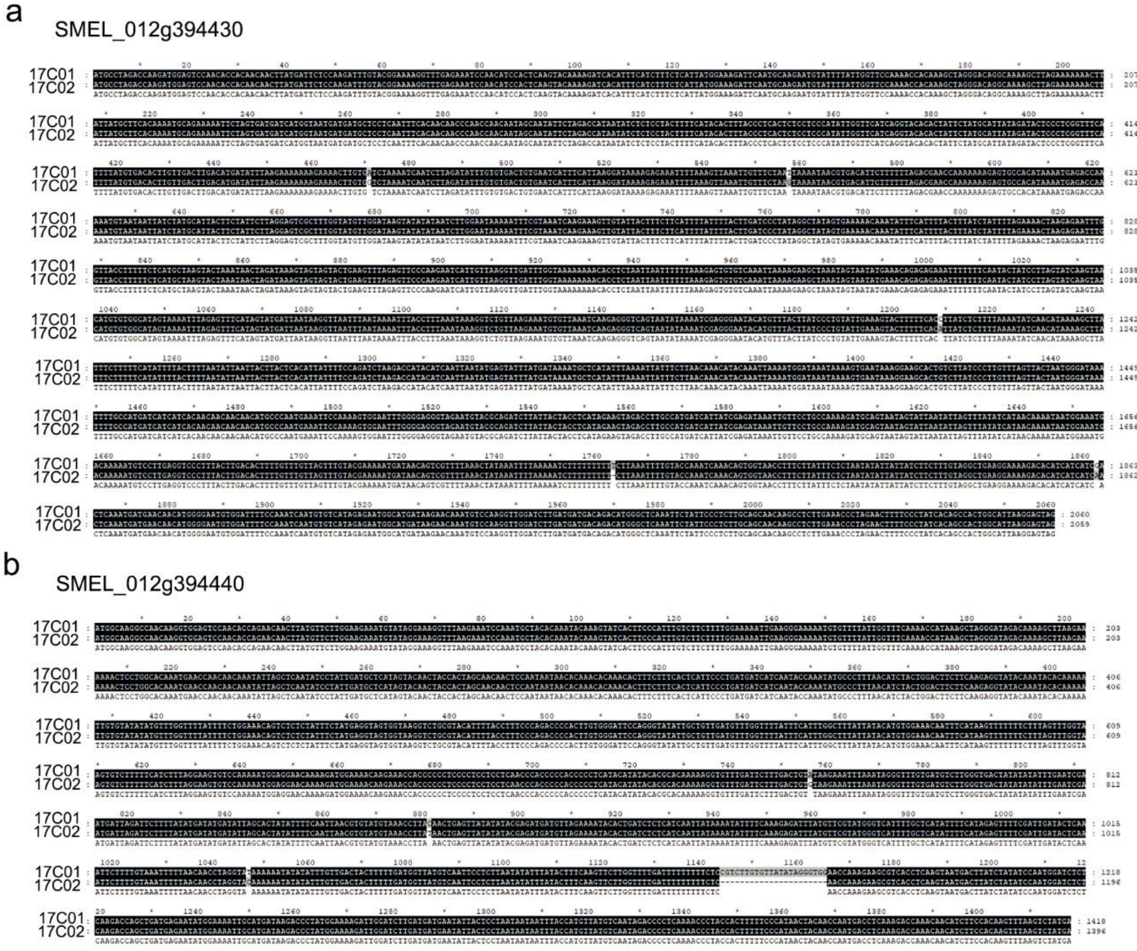

Supplementary Figure 8. The genomic sequence alignments of *SMEL_012g394430* (a) and *SMEL_012g394440* (b).

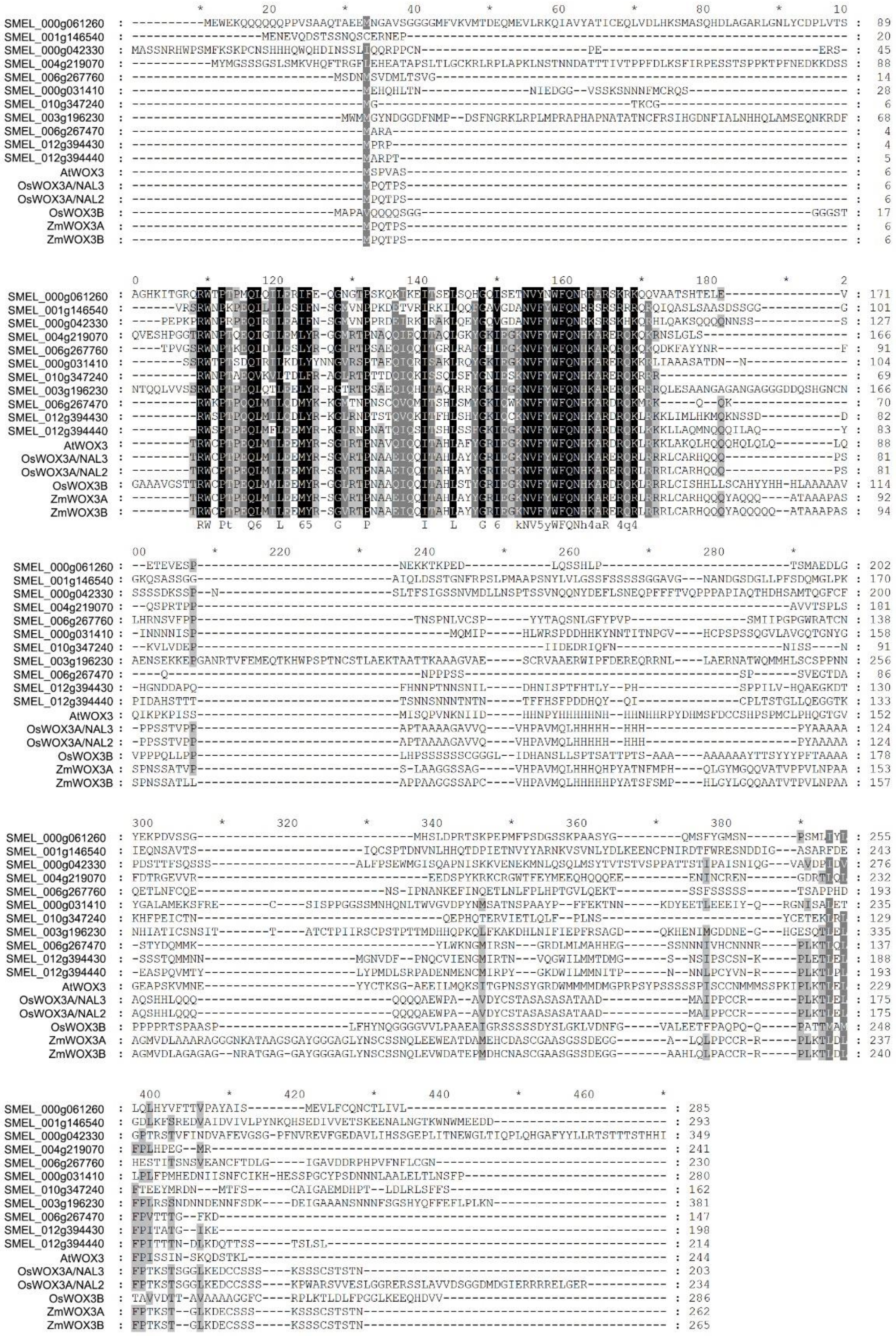

Supplementary Figure 9. The alignment of members in WOX3 group and other SmWUS proteins.

1.1 Supplementary Tables

The Supplementary Tables for this article can be found in the attached file with excel format.
